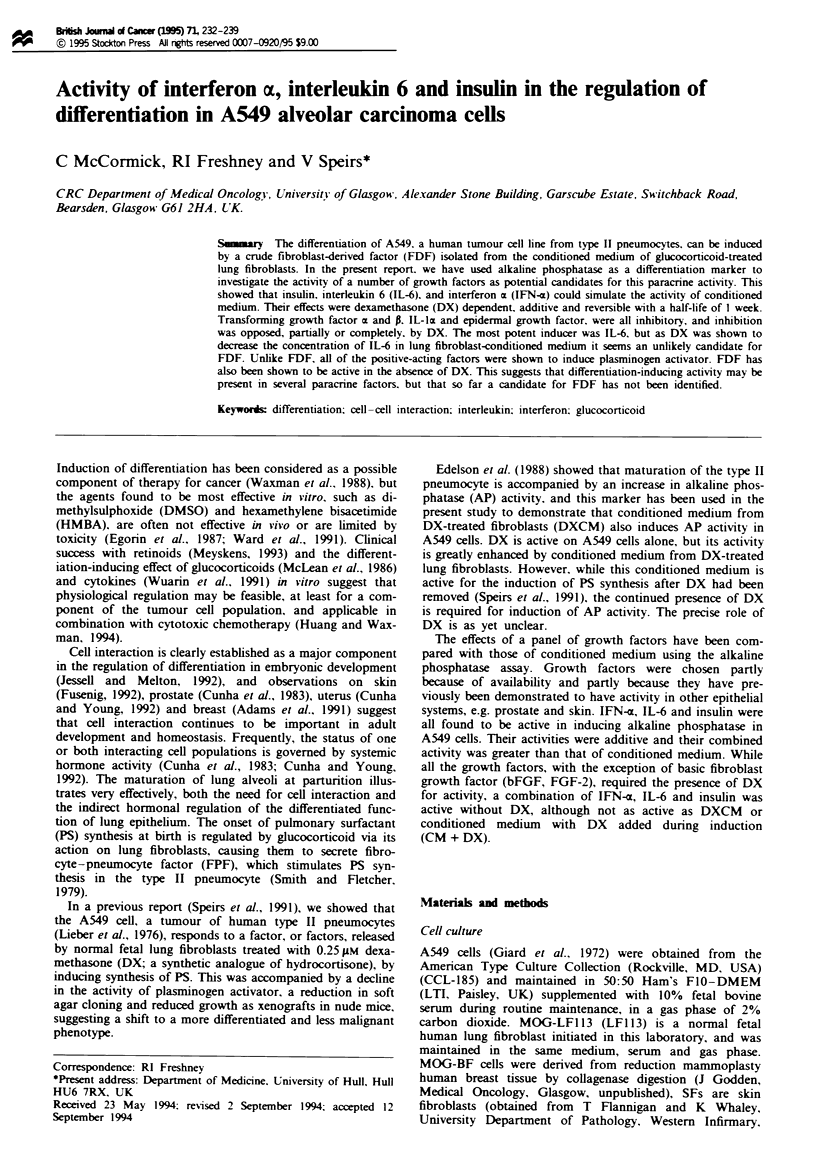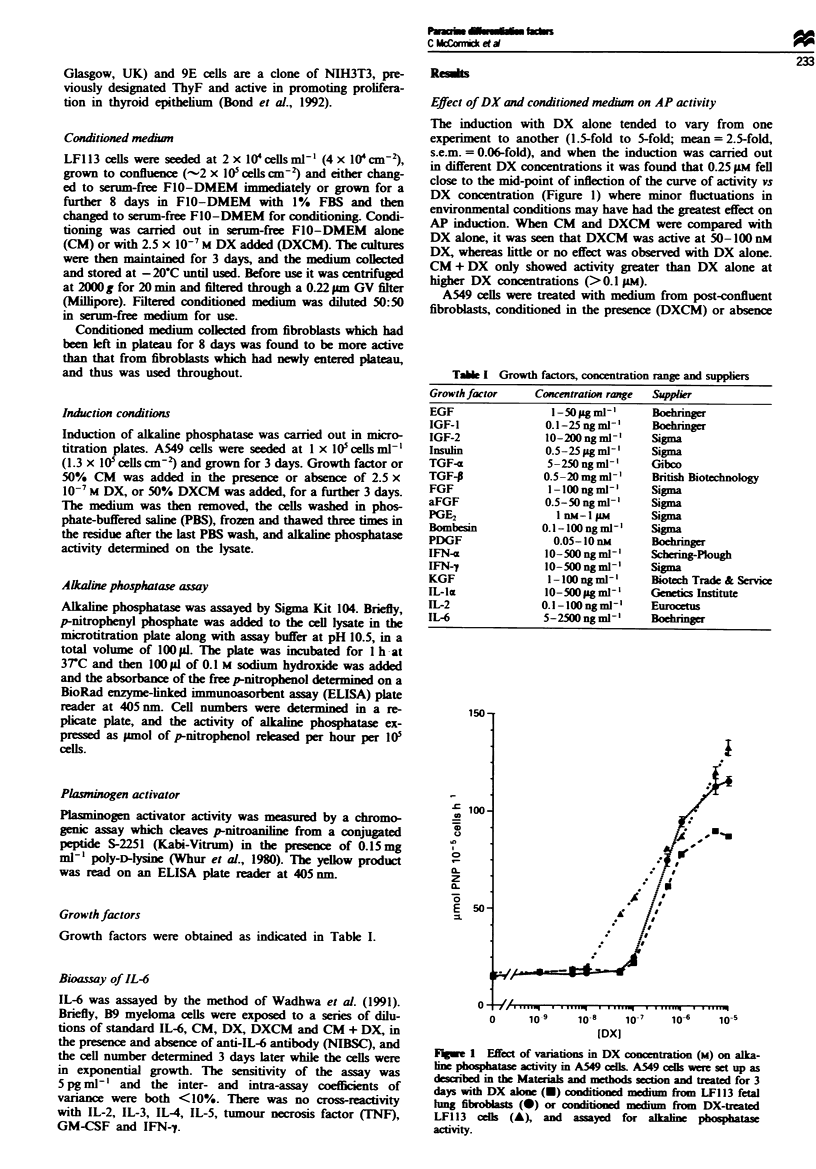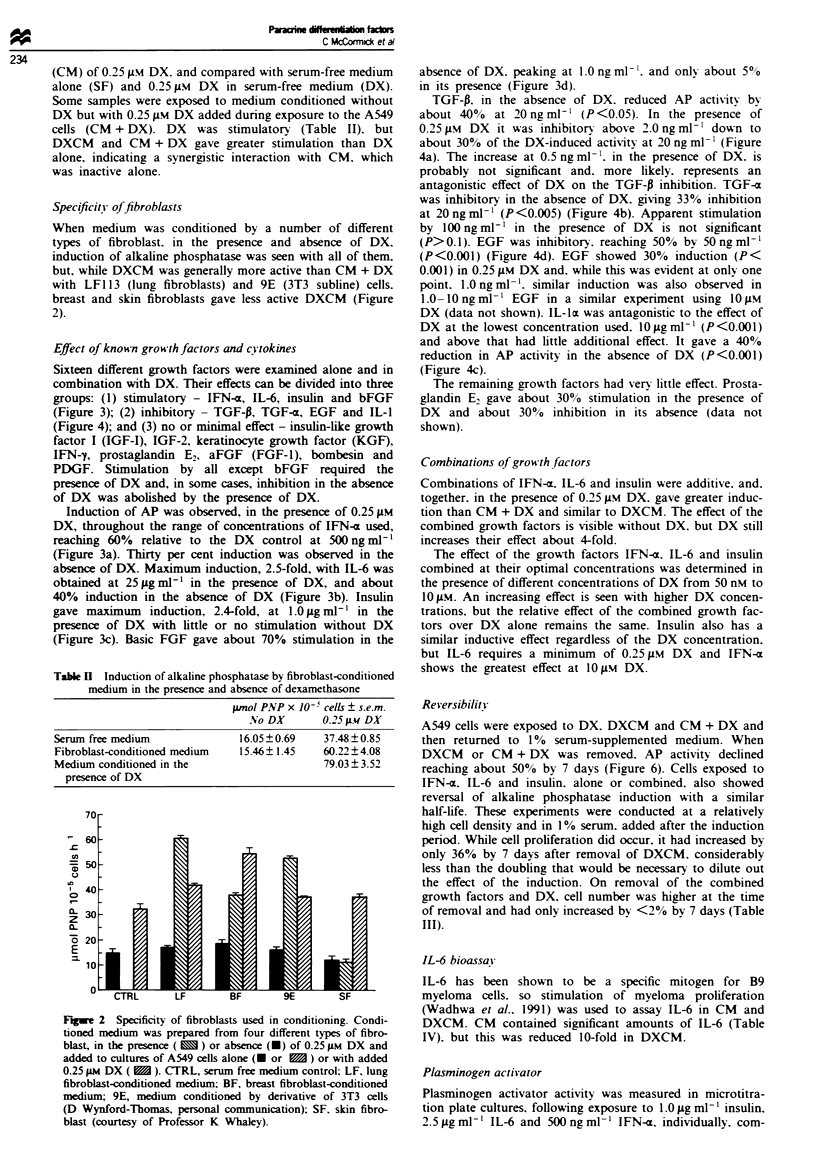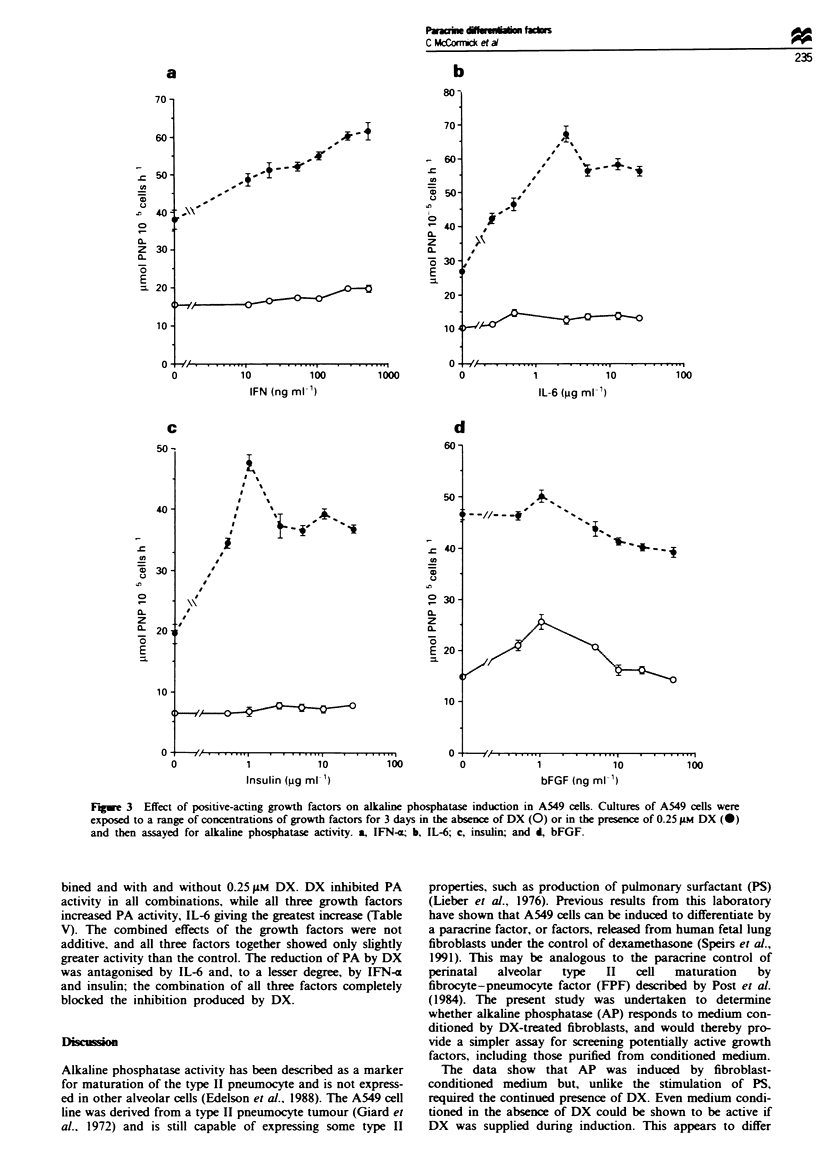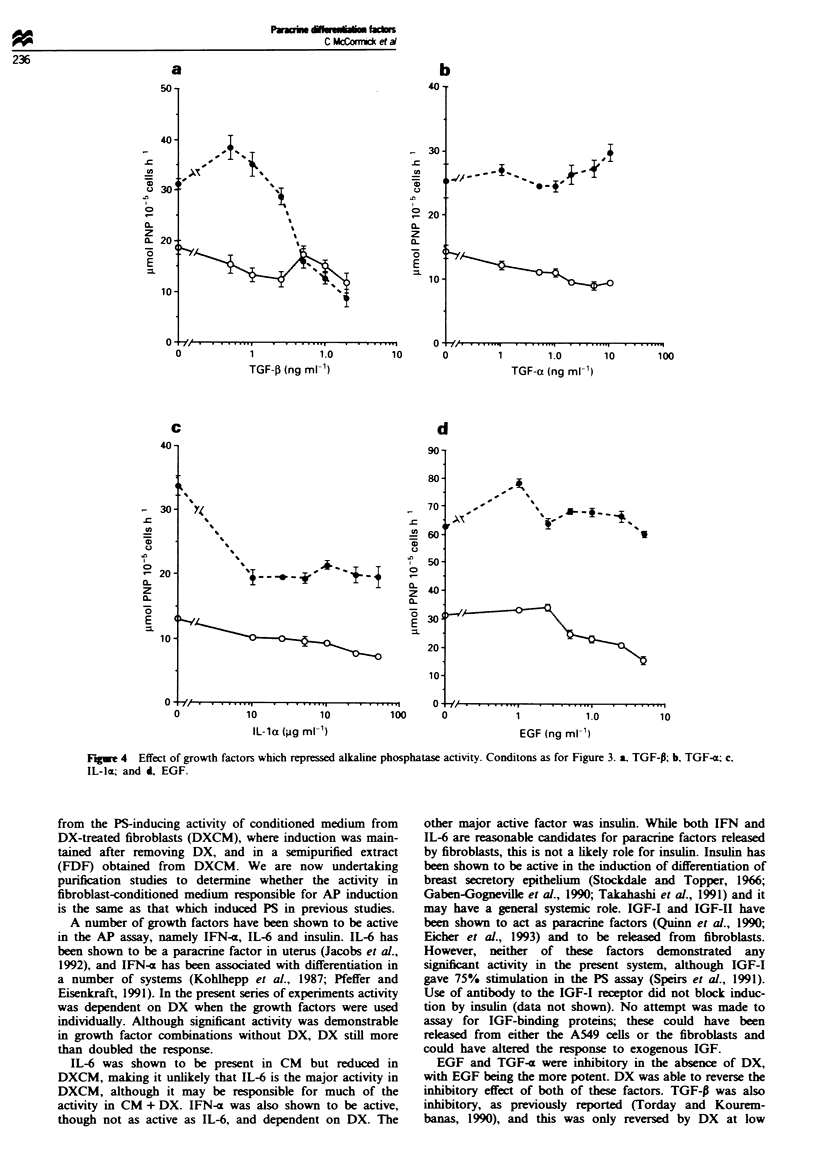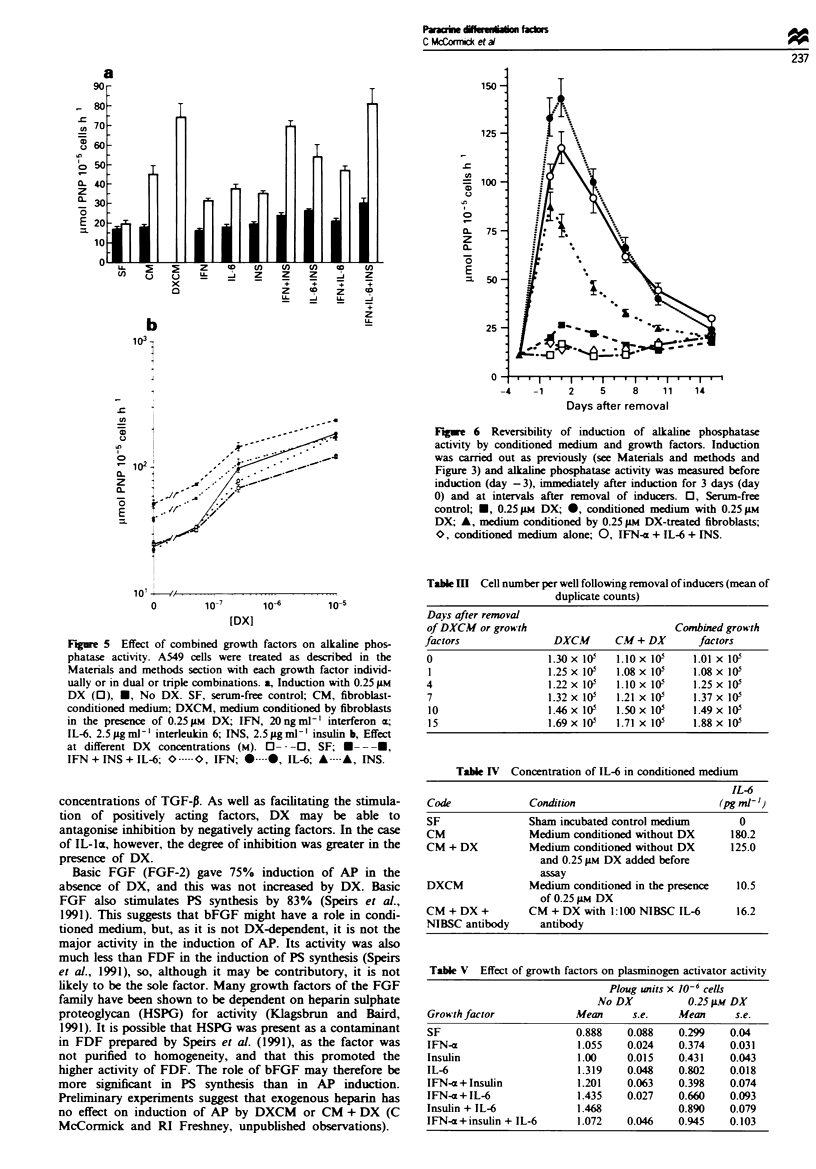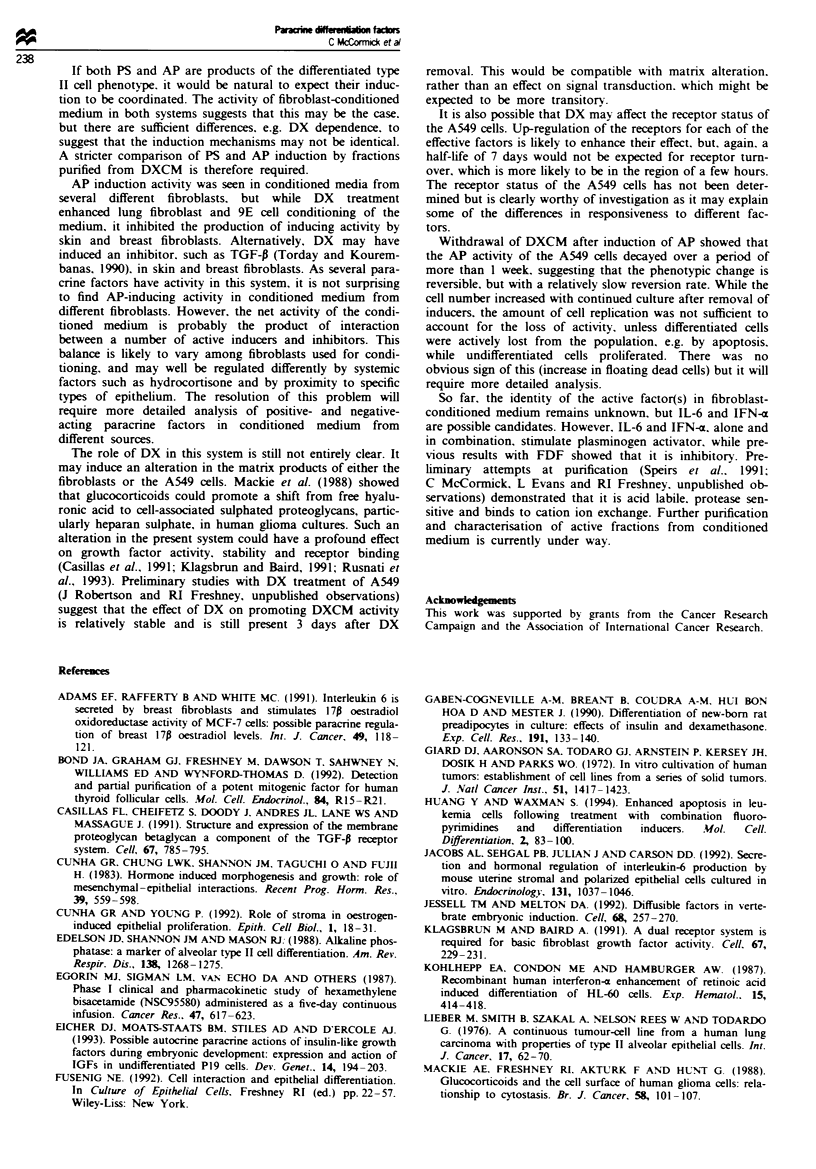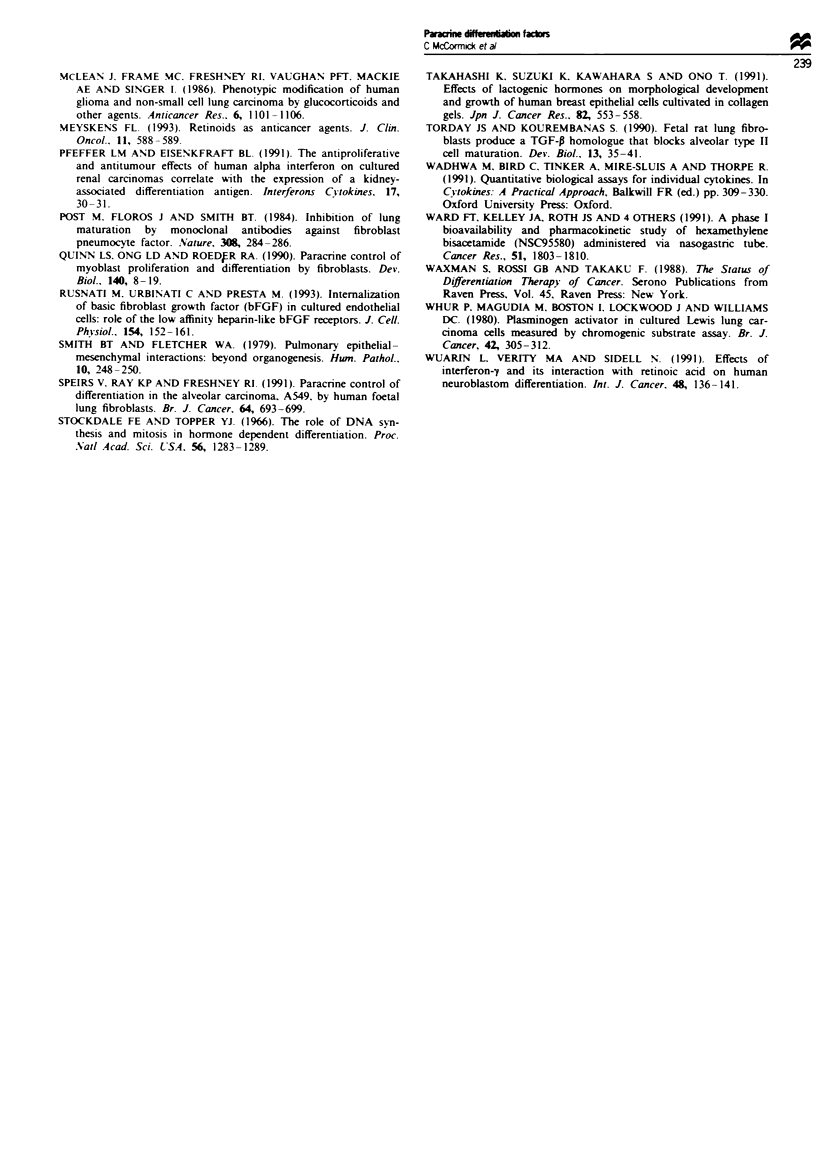# Activity of interferon alpha, interleukin 6 and insulin in the regulation of differentiation in A549 alveolar carcinoma cells.

**DOI:** 10.1038/bjc.1995.49

**Published:** 1995-02

**Authors:** C. McCormick, R. I. Freshney, V. Speirs

**Affiliations:** CRC Department of Medical Oncology, University of Glasgow, Bearsden, UK.

## Abstract

The differentiation of A549, a human tumour cell line from type II pneumocytes, can be induced by a crude fibroblast-derived factor (FDF) isolated from the conditioned medium of glucocorticoid-treated lung fibroblasts. In the present report, we have used alkaline phosphatase as a differentiation marker to investigate the activity of a number of growth factors as potential candidates for this paracrine activity. This showed that insulin, interleukin 6 (IL-6), and interferon alpha (IFN-alpha) could simulate the activity of conditioned medium. Their effects were dexamethasone (DX) dependent, additive and reversible with a half-life of 1 week. Transforming growth factor alpha and beta, IL-1 alpha and epidermal growth factor, were all inhibitory, and inhibition was opposed, partially or completely, by DX. The most potent inducer was IL-6, but as DX was shown to decrease the concentration of IL-6 in lung fibroblast-conditioned medium it seems an unlikely candidate for FDF. Unlike FDF, all of the positive-acting factors were shown to induce plasminogen activator. FDF has also been shown to be active in the absence of DX. This suggests that differentiation-inducing activity may be present in several paracrine factors, but that so far a candidate for FDF has not been identified.


British Journal of Cancer (1995) 71, 232-239

© 1995 Stockton Press All rights reserved 0007-0920/95 $9.00

### Activity of interferon α, interleukin 6 and insulin in the regulation of differentiation in A549 alveolar carcinoma cells

C McCormick, RI Freshney and V Speirs*

*CRC Department of Medical Oncology, University of Glasgow, Alexander Stone Building, Garscube Estate, Switchback Road, Bearsden, Glasgow G61 2HA, UK.*

**Summary** The differentiation of A549, a human tumour cell line from type II pneumocytes, can be induced by a crude fibroblast-derived factor (FDF) isolated from the conditioned medium of glucocorticoid-treated lung fibroblasts. In the present report, we have used alkaline phosphatase as a differentiation marker to investigate the activity of a number of growth factors as potential candidates for this paracrine activity. This showed that insulin, interleukin 6 (IL-6), and interferon α (IFN-α) could simulate the activity of conditioned medium. Their effects were dexamethasone (DX) dependent, additive and reversible with a half-life of 1 week. Transforming growth factor α and β, IL-1α and epidermal growth factor, were all inhibitory, and inhibition was opposed, partially or completely, by DX. The most potent inducer was IL-6, but as DX was shown to decrease the concentration of IL-6 in lung fibroblast-conditioned medium it seems an unlikely candidate for FDF. Unlike FDF, all of the positive-acting factors were shown to induce plasminogen activator. FDF has also been shown to be active in the absence of DX. This suggests that differentiation-inducing activity may be present in several paracrine factors, but that so far a candidate for FDF has not been identified.

**Keywords:** differentiation; cell-cell interaction; interleukin; interferon; glucocorticoid

Induction of differentiation has been considered as a possible component of therapy for cancer (Waxman *et al.*, 1988), but the agents found to be most effective *in vitro*, such as dimethylsulphoxide (DMSO) and hexamethylene bisacetimide (HMBA), are often not effective *in vivo* or are limited by toxicity (Egorin *et al.*, 1987; Ward *et al.*, 1991). Clinical success with retinoids (Meyskens, 1993) and the differentiation-inducing effect of glucocorticoids (McLean *et al.*, 1986) and cytokines (Wuarin *et al.*, 1991) *in vitro* suggest that physiological regulation may be feasible, at least for a component of the tumour cell population, and applicable in combination with cytotoxic chemotherapy (Huang and Waxman, 1994).

Cell interaction is clearly established as a major component in the regulation of differentiation in embryonic development (Jessell and Melton, 1992), and observations on skin (Fusenig, 1992), prostate (Cunha *et al.*, 1983), uterus (Cunha and Young, 1992) and breast (Adams *et al.*, 1991) suggest that cell interaction continues to be important in adult development and homeostasis. Frequently, the status of one or both interacting cell populations is governed by systemic hormone activity (Cunha *et al.*, 1983; Cunha and Young, 1992). The maturation of lung alveoli at parturition illustrates very effectively, both the need for cell interaction and the indirect hormonal regulation of the differentiated function of lung epithelium. The onset of pulmonary surfactant (PS) synthesis at birth is regulated by glucocorticoid via its action on lung fibroblasts, causing them to secrete fibrocyte-pneumocyte factor (FPF), which stimulates PS synthesis in the type II pneumocyte (Smith and Fletcher, 1979).

In a previous report (Speirs *et al.*, 1991), we showed that the A549 cell, a tumour of human type II pneumocytes (Lieber *et al.*, 1976), responds to a factor, or factors, released by normal fetal lung fibroblasts treated with 0.25 μM dexamethasone (DX; a synthetic analogue of hydrocortisone), by inducing synthesis of PS. This was accompanied by a decline in the activity of plasminogen activator, a reduction in soft agar cloning and reduced growth as xenografts in nude mice, suggesting a shift to a more differentiated and less malignant phenotype.

Correspondence: RI Freshney
*Present address: Department of Medicine, University of Hull, Hull HU6 7RX, UK
Received 23 May 1994; revised 2 September 1994; accepted 12 September 1994

Edelson *et al.* (1988) showed that maturation of the type II pneumocyte is accompanied by an increase in alkaline phosphatase (AP) activity, and this marker has been used in the present study to demonstrate that conditioned medium from DX-treated fibroblasts (DXCM) also induces AP activity in A549 cells. DX is active on A549 cells alone, but its activity is greatly enhanced by conditioned medium from DX-treated lung fibroblasts. However, while this conditioned medium is active for the induction of PS synthesis after DX had been removed (Speirs *et al.*, 1991), the continued presence of DX is required for induction of AP activity. The precise role of DX is as yet unclear.

The effects of a panel of growth factors have been compared with those of conditioned medium using the alkaline phosphatase assay. Growth factors were chosen partly because of availability and partly because they have previously been demonstrated to have activity in other epithelial systems, e.g. prostate and skin. IFN-α, IL-6 and insulin were all found to be active in inducing alkaline phosphatase in A549 cells. Their activities were additive and their combined activity was greater than that of conditioned medium. While all the growth factors, with the exception of basic fibroblast growth factor (bFGF, FGF-2), required the presence of DX for activity, a combination of IFN-α, IL-6 and insulin was active without DX, although not as active as DXCM or conditioned medium with DX added during induction (CM + DX).

### Materials and methods

### Cell culture

A549 cells (Giard *et al.*, 1972) were obtained from the American Type Culture Collection (Rockville, MD, USA) (CCL-185) and maintained in 50:50 Ham's F10-DMEM (LTI, Paisley, UK) supplemented with 10% fetal bovine serum during routine maintenance, in a gas phase of 2% carbon dioxide. MOG-LF113 (LF113) is a normal fetal human lung fibroblast initiated in this laboratory, and was maintained in the same medium, serum and gas phase. MOG-BF cells were derived from reduction mammoplasty human breast tissue by collagenase digestion (J Godden, Medical Oncology, Glasgow, unpublished). SFs are skin fibroblasts (obtained from T Flannigan and K Whaley, University Department of Pathology, Western Infirmary,

Glasgow, UK) and 9E cells are a clone of NIH3T3, previously designated ThyF and active in promoting proliferation in thyroid epithelium (Bond *et al*., 1992).

### *Conditioned medium*

LF113 cells were seeded at $2 \times 10^4$ cells ml$^{-1}$ ($4 \times 10^4$ cm$^{-2}$), grown to confluence ($\sim 2 \times 10^5$ cells cm$^{-2}$) and either changed to serum-free F10-DMEM immediately or grown for a further 8 days in F10-DMEM with 1% FBS and then changed to serum-free F10-DMEM for conditioning. Conditioning was carried out in serum-free F10-DMEM alone (CM) or with $2.5 \times 10^{-7}$ M DX added (DXCM). The cultures were then maintained for 3 days, and the medium collected and stored at $-20$°C until used. Before use it was centrifuged at 2000 *g* for 20 min and filtered through a 0.22 μm GV filter (Millipore). Filtered conditioned medium was diluted 50:50 in serum-free medium for use.

Conditioned medium collected from fibroblasts which had been left in plateau for 8 days was found to be more active than that from fibroblasts which had newly entered plateau, and thus was used throughout.

### *Induction conditions*

Induction of alkaline phosphatase was carried out in microtitration plates. A549 cells were seeded at $1 \times 10^5$ cells ml$^{-1}$ ($1.3 \times 10^5$ cells cm$^{-2}$) and grown for 3 days. Growth factor or 50% CM was added in the presence or absence of $2.5 \times 10^{-7}$ M DX, or 50% DXCM was added, for a further 3 days. The medium was then removed, the cells washed in phosphate-buffered saline (PBS), frozen and thawed three times in the residue after the last PBS wash, and alkaline phosphatase activity determined on the lysate.

### *Alkaline phosphatase assay*

Alkaline phosphatase was assayed by Sigma Kit 104. Briefly, *p*-nitrophenyl phosphate was added to the cell lysate in the microtitration plate along with assay buffer at pH 10.5, in a total volume of 100 μl. The plate was incubated for 1 h at 37°C and then 100 μl of 0.1 M sodium hydroxide was added and the absorbance of the free *p*-nitrophenol determined on a BioRad enzyme-linked immunoasorbent assay (ELISA) plate reader at 405 nm. Cell numbers were determined in a replicate plate, and the activity of alkaline phosphatase expressed as μmol of *p*-nitrophenol released per hour per $10^5$ cells.

### *Plasminogen activator*

Plasminogen activator activity was measured by a chromogenic assay which cleaves *p*-nitroaniline from a conjugated peptide S-2251 (Kabi-Vitrum) in the presence of 0.15 mg ml$^{-1}$ poly-D-lysine (Whur *et al*., 1980). The yellow product was read on an ELISA plate reader at 405 nm.

### *Growth factors*

Growth factors were obtained as indicated in Table I.

### *Bioassay of IL-6*

IL-6 was assayed by the method of Wadhwa *et al*. (1991). Briefly, B9 myeloma cells were exposed to a series of dilutions of standard IL-6, CM, DX, DXCM and CM + DX, in the presence and absence of anti-IL-6 antibody (NIBSC), and the cell number determined 3 days later while the cells were in exponential growth. The sensitivity of the assay was 5 pg ml$^{-1}$ and the inter- and intra-assay coefficients of variance were both <10%. There was no cross-reactivity with IL-2, IL-3, IL-4, IL-5, tumour necrosis factor (TNF), GM-CSF and IFN-γ.

### Results

### *Effect of DX and conditioned medium on AP activity*

The induction with DX alone tended to vary from one experiment to another (1.5-fold to 5-fold; mean = 2.5-fold, s.e.m. = 0.06-fold), and when the induction was carried out in different DX concentrations it was found that 0.25 μM fell close to the mid-point of inflection of the curve of activity *vs* DX concentration (Figure 1) where minor fluctuations in environmental conditions may have had the greatest effect on AP induction. When CM and DXCM were compared with DX alone, it was seen that DXCM was active at 50–100 nM DX, whereas little or no effect was observed with DX alone. CM + DX only showed activity greater than DX alone at higher DX concentrations (>0.1 μM).

A549 cells were treated with medium from post-confluent fibroblasts, conditioned in the presence (DXCM) or absence

**Table I** Growth factors, concentration range and suppliers

| *Growth factor* | *Concentration range* | *Supplier* |
|---|---|---|
| EGF | 1–50 μg ml$^{-1}$ | Boehringer |
| IGF-1 | 0.1–25 ng ml$^{-1}$ | Boehringer |
| IGF-2 | 10–200 ng ml$^{-1}$ | Sigma |
| Insulin | 0.5–25 μg ml$^{-1}$ | Sigma |
| TGF-α | 5–250 ng ml$^{-1}$ | Gibco |
| TGF-β | 0.5–20 mg ml$^{-1}$ | British Biotechnology |
| FGF | 1–100 ng ml$^{-1}$ | Sigma |
| aFGF | 0.5–50 ng ml$^{-1}$ | Sigma |
| $PGE_2$ | 1 nM–1 μM | Sigma |
| Bombesin | 0.1–100 ng ml$^{-1}$ | Sigma |
| PDGF | 0.05–10 nM | Boehringer |
| IFN-α | 10–500 ng ml$^{-1}$ | Schering-Plough |
| IFN-γ | 10–500 ng ml$^{-1}$ | Sigma |
| KGF | 1–100 ng ml$^{-1}$ | Biotech Trade & Service |
| IL-1α | 10–500 μg ml$^{-1}$ | Genetics Institute |
| IL-2 | 0.1–100 ng ml$^{-1}$ | Eurocetus |
| IL-6 | 5–2500 ng ml$^{-1}$ | Boehringer |

**Figure 1** Effect of variations in DX concentration (M) on alkaline phosphatase activity in A549 cells. A549 cells were set up as described in the Materials and methods section and treated for 3 days with DX alone (■) conditioned medium from LF113 fetal lung fibroblasts (●) or conditioned medium from DX-treated LF113 cells (▲), and assayed for alkaline phosphatase activity.

(CM) of 0.25 μM DX, and compared with serum-free medium alone (SF) and 0.25 μM DX in serum-free medium (DX). Some samples were exposed to medium conditioned without DX but with 0.25 μM DX added during exposure to the A549 cells (CM + DX). DX was stimulatory (Table II), but DXCM and CM + DX gave greater stimulation than DX alone, indicating a synergistic interaction with CM, which was inactive alone.

*Specificity of fibroblasts*

When medium was conditioned by a number of different types of fibroblast, in the presence and absence of DX, induction of alkaline phosphatase was seen with all of them, but, while DXCM was generally more active than CM + DX with LF113 (lung fibroblasts) and 9E (3T3 subline) cells, breast and skin fibroblasts gave less active DXCM (Figure 2).

*Effect of known growth factors and cytokines*

Sixteen different growth factors were examined alone and in combination with DX. Their effects can be divided into three groups: (1) stimulatory - IFN-α, IL-6, insulin and bFGF (Figure 3); (2) inhibitory - TGF-β, TGF-α, EGF and IL-1 (Figure 4); and (3) no or minimal effect - insulin-like growth factor I (IGF-I), IGF-2, keratinocyte growth factor (KGF), IFN-γ, prostaglandin $E_2$, aFGF (FGF-1), bombesin and PDGF. Stimulation by all except bFGF required the presence of DX and, in some cases, inhibition in the absence of DX was abolished by the presence of DX.

Induction of AP was observed, in the presence of 0.25 μM DX, throughout the range of concentrations of IFN-α used, reaching 60% relative to the DX control at 500 ng $ml^{-1}$ (Figure 3a). Thirty per cent induction was observed in the absence of DX. Maximum induction, 2.5-fold, with IL-6 was obtained at 25 μg $ml^{-1}$ in the presence of DX, and about 40% induction in the absence of DX (Figure 3b). Insulin gave maximum induction, 2.4-fold, at 1.0 μg $ml^{-1}$ in the presence of DX with little or no stimulation without DX (Figure 3c). Basic FGF gave about 70% stimulation in the absence of DX, peaking at 1.0 ng $ml^{-1}$, and only about 5% in its presence (Figure 3d).

TGF-β, in the absence of DX, reduced AP activity by about 40% at 20 ng $ml^{-1}$ ($P<0.05$). In the presence of 0.25 μM DX it was inhibitory above 2.0 ng $ml^{-1}$ down to about 30% of the DX-induced activity at 20 ng $ml^{-1}$ (Figure 4a). The increase at 0.5 ng $ml^{-1}$, in the presence of DX, is probably not significant and, more likely, represents an antagonistic effect of DX on the TGF-β inhibition. TGF-α was inhibitory in the absence of DX, giving 33% inhibition at 20 ng $ml^{-1}$ ($P<0.005$) (Figure 4b). Apparent stimulation by 100 ng $ml^{-1}$ in the presence of DX is not significant ($P>0.1$). EGF was inhibitory, reaching 50% by 50 ng $ml^{-1}$ ($P<0.001$) (Figure 4d). EGF showed 30% induction ($P<0.001$) in 0.25 μM DX and, while this was evident at only one point, 1.0 ng $ml^{-1}$, similar induction was also observed in 1.0-10 ng $ml^{-1}$ EGF in a similar experiment using 10 μM DX (data not shown). IL-1α was antagonistic to the effect of DX at the lowest concentration used, 10 μg $ml^{-1}$ ($P<0.001$) and above that had little additional effect. It gave a 40% reduction in AP activity in the absence of DX ($P<0.001$) (Figure 4c).

The remaining growth factors had very little effect. Prostaglandin $E_2$ gave about 30% stimulation in the presence of DX and about 30% inhibition in its absence (data not shown).

*Combinations of growth factors*

Combinations of IFN-α, IL-6 and insulin were additive, and, together, in the presence of 0.25 μM DX, gave greater induction than CM + DX and similar to DXCM. The effect of the combined growth factors is visible without DX, but DX still increases their effect about 4-fold.

The effect of the growth factors IFN-α, IL-6 and insulin combined at their optimal concentrations was determined in the presence of different concentrations of DX from 50 nM to 10 μM. An increasing effect is seen with higher DX concentrations, but the relative effect of the combined growth factors over DX alone remains the same. Insulin also has a similar inductive effect regardless of the DX concentration, but IL-6 requires a minimum of 0.25 μM DX and IFN-α shows the greatest effect at 10 μM DX.

*Reversibility*

A549 cells were exposed to DX, DXCM and CM + DX and then returned to 1% serum-supplemented medium. When DXCM or CM + DX was removed, AP activity declined reaching about 50% by 7 days (Figure 6). Cells exposed to IFN-α, IL-6 and insulin, alone or combined, also showed reversal of alkaline phosphatase induction with a similar half-life. These experiments were conducted at a relatively high cell density and in 1% serum, added after the induction period. While cell proliferation did occur, it had increased by only 36% by 7 days after removal of DXCM, considerably less than the doubling that would be necessary to dilute out the effect of the induction. On removal of the combined growth factors and DX, cell number was higher at the time of removal and had only increased by <2% by 7 days (Table III).

*IL-6 bioassay*

IL-6 has been shown to be a specific mitogen for B9 myeloma cells, so stimulation of myeloma proliferation (Wadhwa *et al.*, 1991) was used to assay IL-6 in CM and DXCM. CM contained significant amounts of IL-6 (Table IV), but this was reduced 10-fold in DXCM.

*Plasminogen activator*

Plasminogen activator activity was measured in microtitration plate cultures, following exposure to 1.0 μg $ml^{-1}$ insulin, 2.5 μg $ml^{-1}$ IL-6 and 500 ng $ml^{-1}$ IFN-α, individually, com-

**Table II** Induction of alkaline phosphatase by fibroblast-conditioned medium in the presence and absence of dexamethasone

| | *μmol PNP × $10^{-5}$ cells ± s.e.m.* No DX | *0.25 μM DX* |
|---|---|---|
| Serum free medium | 16.05±0.69 | 37.48±0.85 |
| Fibroblast-conditioned medium | 15.46±1.45 | 60.22±4.08 |
| Medium conditioned in the presence of DX | | 79.03±3.52 |

**Figure 2** Specificity of fibroblasts used in conditioning. Conditioned medium was prepared from four different types of fibroblast, in the presence ( ▧ ) or absence (■) of 0.25 μM DX and added to cultures of A549 cells alone (■ or ▨ ) or with added 0.25 μM DX ( ▨ ). CTRL, serum free medium control; LF, lung fibroblast-conditioned medium; BF, breast fibroblast-conditioned medium; 9E, medium conditioned by derivative of 3T3 cells (D Wynford-Thomas, personal communication); SF, skin fibroblast (courtesy of Professor K Whaley).

**Figure 3** Effect of positive-acting growth factors on alkaline phosphatase induction in A549 cells. Cultures of A549 cells were exposed to a range of concentrations of growth factors for 3 days in the absence of DX (○) or in the presence of 0.25 μM DX (●) and then assayed for alkaline phosphatase activity. **a**, IFN-α; **b**, IL-6; **c**, insulin; and **d**, bFGF.

bined and with and without 0.25 μM DX. DX inhibited PA activity in all combinations, while all three growth factors increased PA activity, IL-6 giving the greatest increase (Table V). The combined effects of the growth factors were not additive, and all three factors together showed only slightly greater activity than the control. The reduction of PA by DX was antagonised by IL-6 and, to a lesser degree, by IFN-α and insulin; the combination of all three factors completely blocked the inhibition produced by DX.

### Discussion

Alkaline phosphatase activity has been described as a marker for maturation of the type II pneumocyte and is not expressed in other alveolar cells (Edelson *et al.*, 1988). The A549 cell line was derived from a type II pneumocyte tumour (Giard *et al.*, 1972) and is still capable of expressing some type II properties, such as production of pulmonary surfactant (PS) (Lieber *et al.*, 1976). Previous results from this laboratory have shown that A549 cells can be induced to differentiate by a paracrine factor, or factors, released from human fetal lung fibroblasts under the control of dexamethasone (Speirs *et al.*, 1991). This may be analogous to the paracrine control of perinatal alveolar type II cell maturation by fibrocyte–pneumocyte factor (FPF) described by Post *et al.* (1984). The present study was undertaken to determine whether alkaline phosphatase (AP) responds to medium conditioned by DX-treated fibroblasts, and would thereby provide a simpler assay for screening potentially active growth factors, including those purified from conditioned medium.

The data show that AP was induced by fibroblast-conditioned medium but, unlike the stimulation of PS, required the continued presence of DX. Even medium conditioned in the absence of DX could be shown to be active if DX was supplied during induction. This appears to differ

**Figure 4** Effect of growth factors which repressed alkaline phosphatase activity. Conditons as for Figure 3. **a**, TGF-β; **b**, TGF-α; **c**, IL-1α; and **d**, EGF.

from the PS-inducing activity of conditioned medium from DX-treated fibroblasts (DXCM), where induction was maintained after removing DX, and in a semipurified extract (FDF) obtained from DXCM. We are now undertaking purification studies to determine whether the activity in fibroblast-conditioned medium responsible for AP induction is the same as that which induced PS in previous studies.

A number of growth factors have been shown to be active in the AP assay, namely IFN-α, IL-6 and insulin. IL-6 has been shown to be a paracrine factor in uterus (Jacobs *et al.*, 1992), and IFN-α has been associated with differentiation in a number of systems (Kohlhepp *et al.*, 1987; Pfeffer and Eisenkraft, 1991). In the present series of experiments activity was dependent on DX when the growth factors were used individually. Although significant activity was demonstrable in growth factor combinations without DX, DX still more than doubled the response.

IL-6 was shown to be present in CM but reduced in DXCM, making it unlikely that IL-6 is the major activity in DXCM, although it may be responsible for much of the activity in CM + DX. IFN-α was also shown to be active, though not as active as IL-6, and dependent on DX. The other major active factor was insulin. While both IFN and IL-6 are reasonable candidates for paracrine factors released by fibroblasts, this is not a likely role for insulin. Insulin has been shown to be active in the induction of differentiation of breast secretory epithelium (Stockdale and Topper, 1966; Gaben-Gogneville *et al.*, 1990; Takahashi *et al.*, 1991) and it may have a general systemic role. IGF-I and IGF-II have been shown to act as paracrine factors (Quinn *et al.*, 1990; Eicher *et al.*, 1993) and to be released from fibroblasts. However, neither of these factors demonstrated any significant activity in the present system, although IGF-I gave 75% stimulation in the PS assay (Speirs *et al.*, 1991). Use of antibody to the IGF-I receptor did not block induction by insulin (data not shown). No attempt was made to assay for IGF-binding proteins; these could have been released from either the A549 cells or the fibroblasts and could have altered the response to exogenous IGF.

EGF and TGF-α were inhibitory in the absence of DX, with EGF being the more potent. DX was able to reverse the inhibitory effect of both of these factors. TGF-β was also inhibitory, as previously reported (Torday and Kourembanas, 1990), and this was only reversed by DX at low

**Figure 5** Effect of combined growth factors on alkaline phosphatase activity. A549 cells were treated as described in the Materials and methods section with each growth factor individually or in dual or triple combinations. **a**, Induction with 0.25 μM DX (□). ■, No DX. SF, serum-free control; CM, fibroblast-conditioned medium; DXCM, medium conditioned by fibroblasts in the presence of 0.25 μM DX; IFN, 20 ng $ml^{-1}$ interferon α; IL-6, 2.5 μg $ml^{-1}$ interleukin 6; INS, 2.5 μg $ml^{-1}$ insulin **b**, Effect at different DX concentrations (M). □- · -□, SF; ■- - -■, IFN + INS + IL-6; ◇·····◇, IFN; ●····●, IL-6; ▲····▲, INS.

**Figure 6** Reversibility of induction of alkaline phosphatase activity by conditioned medium and growth factors. Induction was carried out as previously (see Materials and methods and Figure 3) and alkaline phosphatase activity was measured before induction (day −3), immediately after induction for 3 days (day 0) and at intervals after removal of inducers. □, Serum-free control; ■, 0.25 μM DX; ●, conditioned medium with 0.25 μM DX; ▲, medium conditioned by 0.25 μM DX-treated fibroblasts; ◇, conditioned medium alone; O, IFN-α + IL-6 + INS.

concentrations of TGF-β. As well as facilitating the stimulation of positively acting factors, DX may be able to antagonise inhibition by negatively acting factors. In the case of IL-1α, however, the degree of inhibition was greater in the presence of DX.

Basic FGF (FGF-2) gave 75% induction of AP in the absence of DX, and this was not increased by DX. Basic FGF also stimulates PS synthesis by 83% (Speirs *et al.*, 1991). This suggests that bFGF might have a role in conditioned medium, but, as it is not DX-dependent, it is not the major activity in the induction of AP. Its activity was also much less than FDF in the induction of PS synthesis (Speirs *et al.*, 1991), so, although it may be contributory, it is not likely to be the sole factor. Many growth factors of the FGF family have been shown to be dependent on heparin sulphate proteoglycan (HSPG) for activity (Klagsbrun and Baird, 1991). It is possible that HSPG was present as a contaminant in FDF prepared by Speirs *et al.* (1991), as the factor was not purified to homogeneity, and that this promoted the higher activity of FDF. The role of bFGF may therefore be more significant in PS synthesis than in AP induction. Preliminary experiments suggest that exogenous heparin has no effect on induction of AP by DXCM or CM + DX (C McCormick and RI Freshney, unpublished observations).

**Table III** Cell number per well following removal of inducers (mean of duplicate counts)

| *Days after removal of DXCM or growth factors* | *DXCM* | *CM + DX* | *Combined growth factors* |
|---|---|---|---|
| 0 | 1.30 × $10^5$ | 1.10 × $10^5$ | 1.01 × $10^5$ |
| 1 | 1.25 × $10^5$ | 1.08 × $10^5$ | 1.08 × $10^5$ |
| 4 | 1.22 × $10^5$ | 1.10 × $10^5$ | 1.25 × $10^5$ |
| 7 | 1.32 × $10^5$ | 1.21 × $10^5$ | 1.37 × $10^5$ |
| 10 | 1.46 × $10^5$ | 1.50 × $10^5$ | 1.49 × $10^5$ |
| 15 | 1.69 × $10^5$ | 1.71 × $10^5$ | 1.88 × $10^5$ |

**Table IV** Concentration of IL-6 in conditioned medium

| *Code* | *Condition* | *IL-6 (pg $ml^{-1}$)* |
|---|---|---|
| SF | Sham incubated control medium | 0 |
| CM | Medium conditioned without DX | 180.2 |
| CM + DX | Medium conditioned without DX and 0.25 μM DX added before assay | 125.0 |
| DXCM | Medium conditioned in the presence of 0.25 μM DX | 10.5 |
| CM + DX + NIBSC antibody | CM + DX with 1:100 NIBSC IL-6 antibody | 16.2 |

**Table V** Effect of growth factors on plasminogen activator activity

| | *Ploug units × $10^{-6}$ cells* | | | |
|---|---|---|---|---|
| | *No DX* | | *0.25 μM DX* | |
| *Growth factor* | *Mean* | *s.e.* | *Mean* | *s.e.* |
| SF | 0.888 | 0.088 | 0.299 | 0.04 |
| IFN-α | 1.055 | 0.024 | 0.374 | 0.031 |
| Insulin | 1.00 | 0.015 | 0.431 | 0.043 |
| IL-6 | 1.319 | 0.048 | 0.802 | 0.018 |
| IFN-α + Insulin | 1.201 | 0.063 | 0.398 | 0.074 |
| IFN-α + IL-6 | 1.435 | 0.027 | 0.660 | 0.093 |
| Insulin + IL-6 | 1.468 | | 0.890 | 0.079 |
| IFN-α + insulin + IL-6 | 1.072 | 0.046 | 0.945 | 0.103 |

If both PS and AP are products of the differentiated type II cell phenotype, it would be natural to expect their induction to be coordinated. The activity of fibroblast-conditioned medium in both systems suggests that this may be the case, but there are sufficient differences, e.g. DX dependence, to suggest that the induction mechanisms may not be identical. A stricter comparison of PS and AP induction by fractions purified from DXCM is therefore required.

AP induction activity was seen in conditioned media from several different fibroblasts, but while DX treatment enhanced lung fibroblast and 9E cell conditioning of the medium, it inhibited the production of inducing activity by skin and breast fibroblasts. Alternatively, DX may have induced an inhibitor, such as TGF-$\beta$ (Torday and Kourembanas, 1990), in skin and breast fibroblasts. As several paracrine factors have activity in this system, it is not surprising to find AP-inducing activity in conditioned medium from different fibroblasts. However, the net activity of the conditioned medium is probably the product of interaction between a number of active inducers and inhibitors. This balance is likely to vary among fibroblasts used for conditioning, and may well be regulated differently by systemic factors such as hydrocortisone and by proximity to specific types of epithelium. The resolution of this problem will require more detailed analysis of positive- and negative-acting paracrine factors in conditioned medium from different sources.

The role of DX in this system is still not entirely clear. It may induce an alteration in the matrix products of either the fibroblasts or the A549 cells. Mackie *et al.* (1988) showed that glucocorticoids could promote a shift from free hyaluronic acid to cell-associated sulphated proteoglycans, particularly heparan sulphate, in human glioma cultures. Such an alteration in the present system could have a profound effect on growth factor activity, stability and receptor binding (Casillas *et al.*, 1991; Klagsbrun and Baird, 1991; Rusnati *et al.*, 1993). Preliminary studies with DX treatment of A549 (J Robertson and RI Freshney, unpublished observations) suggest that the effect of DX on promoting DXCM activity is relatively stable and is still present 3 days after DX removal. This would be compatible with matrix alteration, rather than an effect on signal transduction, which might be expected to be more transitory.

It is also possible that DX may affect the receptor status of the A549 cells. Up-regulation of the receptors for each of the effective factors is likely to enhance their effect, but, again, a half-life of 7 days would not be expected for receptor turnover, which is more likely to be in the region of a few hours. The receptor status of the A549 cells has not been determined but is clearly worthy of investigation as it may explain some of the differences in responsiveness to different factors.

Withdrawal of DXCM after induction of AP showed that the AP activity of the A549 cells decayed over a period of more than 1 week, suggesting that the phenotypic change is reversible, but with a relatively slow reversion rate. While the cell number increased with continued culture after removal of inducers, the amount of cell replication was not sufficient to account for the loss of activity, unless differentiated cells were actively lost from the population, e.g. by apoptosis, while undifferentiated cells proliferated. There was no obvious sign of this (increase in floating dead cells) but it will require more detailed analysis.

So far, the identity of the active factor(s) in fibroblast-conditioned medium remains unknown, but IL-6 and IFN-$\alpha$ are possible candidates. However, IL-6 and IFN-$\alpha$, alone and in combination, stimulate plasminogen activator, while previous results with FDF showed that it is inhibitory. Preliminary attempts at purification (Speirs *et al.*, 1991; C McCormick, L Evans and RI Freshney, unpublished observations) demonstrated that it is acid labile, protease sensitive and binds to cation ion exchange. Further purification and characterisation of active fractions from conditioned medium is currently under way.

**Acknowledgements**

This work was supported by grants from the Cancer Research Campaign and the Association of International Cancer Research.

### References

ADAMS EF, RAFFERTY B AND WHITE MC. (1991). Interleukin 6 is secreted by breast fibroblasts and stimulates 17$\beta$ oestradiol oxidoreductase activity of MCF-7 cells: possible paracrine regulation of breast 17$\beta$ oestradiol levels. *Int. J. Cancer*, **49**, 118–121.

BOND JA, GRAHAM GJ, FRESHNEY M, DAWSON T, SAHWNEY N, WILLIAMS ED AND WYNFORD-THOMAS D. (1992). Detection and partial purification of a potent mitogenic factor for human thyroid follicular cells. *Mol. Cell. Endocrinol.*, **84**, R15–R21.

CASILLAS FL, CHEIFETZ S, DOODY J, ANDRES JL, LANE WS AND MASSAGUE J. (1991). Structure and expression of the membrane proteoglycan betaglycan a component of the TGF-$\beta$ receptor system. *Cell*, **67**, 785–795.

CUNHA GR, CHUNG LWK, SHANNON JM, TAGUCHI O AND FUJII H. (1983). Hormone induced morphogenesis and growth: role of mesenchymal–epithelial interactions. *Recent Prog. Horm. Res.*, **39**, 559–598.

CUNHA GR AND YOUNG P. (1992). Role of stroma in oestrogen-induced epithelial proliferation. *Epith. Cell Biol.*, **1**, 18–31.

EDELSON JD, SHANNON JM AND MASON RJ. (1988). Alkaline phosphatase: a marker of alveolar type II cell differentiation. *Am. Rev. Respir. Dis.*, **138**, 1268–1275.

EGORIN MJ, SIGMAN LM, VAN ECHO DA AND OTHERS (1987). Phase I clinical and pharmacokinetic study of hexamethylene bisacetamide (NSC95580) administered as a five-day continuous infusion. *Cancer Res.*, **47**, 617–623.

EICHER DJ, MOATS-STAATS BM, STILES AD AND D'ERCOLE AJ. (1993). Possible autocrine paracrine actions of insulin-like growth factors during embryonic development: expression and action of IGFs in undifferentiated P19 cells. *Dev. Genet.*, **14**, 194–203.

FUSENIG NE. (1992). Cell interaction and epithelial differentiation. In *Culture of Epithelial Cells*, Freshney RI (ed.) pp. 22–57. Wiley-Liss: New York.

GABEN-COGNEVILLE A-M, BREANT B, COUDRA A-M, HUI BON HOA D AND MESTER J. (1990). Differentiation of new-born rat preadipocytes in culture: effects of insulin and dexamethasone. *Exp. Cell. Res.*, **191**, 133–140.

GIARD DJ, AARONSON SA, TODARO GJ, ARNSTEIN P, KERSEY JH, DOSIK H AND PARKS WO. (1972). In vitro cultivation of human tumors: establishment of cell lines from a series of solid tumors. *J. Natl Cancer Inst.*, **51**, 1417–1423.

HUANG Y AND WAXMAN S. (1994). Enhanced apoptosis in leukemia cells following treatment with combination fluoropyrimidines and differentiation inducers. *Mol. Cell. Differentiation*, **2**, 83–100.

JACOBS AL, SEHGAL PB, JULIAN J AND CARSON DD. (1992). Secretion and hormonal regulation of interleukin-6 production by mouse uterine stromal and polarized epithelial cells cultured in vitro. *Endocrinology*, **131**, 1037–1046.

JESSELL TM AND MELTON DA. (1992). Diffusible factors in vertebrate embryonic induction. *Cell*, **68**, 257–270.

KLAGSBRUN M AND BAIRD A. (1991). A dual receptor system is required for basic fibroblast growth factor activity. *Cell*, **67**, 229–231.

KOHLHEPP EA, CONDON ME AND HAMBURGER AW. (1987). Recombinant human interferon-$\alpha$ enhancement of retinoic acid induced differentiation of HL-60 cells. *Exp. Hematol.*, **15**, 414–418.

LIEBER M, SMITH B, SZAKAL A, NELSON REES W AND TODARDO G. (1976). A continuous tumour-cell line from a human lung carcinoma with properties of type II alveolar epithelial cells. *Int. J. Cancer*, **17**, 62–70.

MACKIE AE, FRESHNEY RI, AKTURK F AND HUNT G. (1988). Glucocorticoids and the cell surface of human glioma cells: relationship to cytostasis. *Br. J. Cancer*, **58**, 101–107.

MCLEAN J, FRAME MC, FRESHNEY RI, VAUGHAN PFT, MACKIE AE AND SINGER I. (1986). Phenotypic modification of human glioma and non-small cell lung carcinoma by glucocorticoids and other agents. *Anticancer Res.*, **6**, 1101-1106.

MEYSKENS FL. (1993). Retinoids as anticancer agents. *J. Clin. Oncol.*, **11**, 588-589.

PFEFFER LM AND EISENKFRAFT BL. (1991). The antiproliferative and antitumour effects of human alpha interferon on cultured renal carcinomas correlate with the expression of a kidney-associated differentiation antigen. *Interferons Cytokines*, **17**, 30-31.

POST M, FLOROS J AND SMITH BT. (1984). Inhibition of lung maturation by monoclonal antibodies against fibroblast pneumocyte factor. *Nature*, **308**, 284-286.

QUINN LS, ONG LD AND ROEDER RA. (1990). Paracrine control of myoblast proliferation and differentiation by fibroblasts. *Dev. Biol.*, **140**, 8-19.

RUSNATI M, URBINATI C AND PRESTA M. (1993). Internalization of basic fibroblast growth factor (bFGF) in cultured endothelial cells: role of the low affinity heparin-like bFGF receptors. *J. Cell. Physiol.*, **154**, 152-161.

SMITH BT AND FLETCHER WA. (1979). Pulmonary epithelial-mesenchymal interactions: beyond organogenesis. *Hum. Pathol.*, **10**, 248-250.

SPEIRS V, RAY KP AND FRESHNEY RI. (1991). Paracrine control of differentiation in the alveolar carcinoma, A549, by human foetal lung fibroblasts. *Br. J. Cancer*, **64**, 693-699.

STOCKDALE FE AND TOPPER YJ. (1966). The role of DNA synthesis and mitosis in hormone dependent differentiation. *Proc. Natl Acad. Sci. USA*, **56**, 1283-1289.

TAKAHASHI K, SUZUKI K, KAWAHARA S AND ONO T. (1991). Effects of lactogenic hormones on morphological development and growth of human breast epithelial cells cultivated in collagen gels. *Jpn J. Cancer Res.*, **82**, 553-558.

TORDAY JS AND KOUREMBANAS S. (1990). Fetal rat lung fibroblasts produce a TGF-$\beta$ homologue that blocks alveolar type II cell maturation. *Dev. Biol.*, **13**, 35-41.

WADHWA M, BIRD C, TINKER A, MIRE-SLUIS A AND THORPE R. (1991). Quantitative biological assays for individual cytokines. In *Cytokines: A Practical Approach*, Balkwill FR (ed.) pp. 309-330. Oxford University Press: Oxford.

WARD FT, KELLEY JA, ROTH JS AND 4 OTHERS (1991). A phase I bioavailability and pharmacokinetic study of hexamethylene bisacetamide (NSC95580) administered via nasogastric tube. *Cancer Res.*, **51**, 1803-1810.

WAXMAN S, ROSSI GB AND TAKAKU F. (1988). *The Status of Differentiation Therapy of Cancer*. Serono Publications from Raven Press, Vol. 45, Raven Press: New York.

WHUR P, MAGUDIA M, BOSTON I, LOCKWOOD J AND WILLIAMS DC. (1980). Plasminogen activator in cultured Lewis lung carcinoma cells measured by chromogenic substrate assay. *Br. J. Cancer*, **42**, 305-312.

WUARIN L, VERITY MA AND SIDELL N. (1991). Effects of interferon-$\gamma$ and its interaction with retinoic acid on human neuroblastom differentiation. *Int. J. Cancer*, **48**, 136-141.